# Hand-Made Polytetrafluoroethylene Tricuspid-Valved Conduit for Surgical Reconstruction of the Right Ventricular Outflow Tract in a Child With Truncus Arteriosus

**DOI:** 10.7759/cureus.27062

**Published:** 2022-07-20

**Authors:** Vishal V Bhende, Tanishq S Sharma, Hardil P Majmudar, Krishnan Ganapathy Subramaniam, Deepakkumar V Mehta, Amit Kumar, Purvi R Patel, Gurpreet Panesar, Kunal Soni, Kartik B Dhami, Nirja Patel, Sohilkhan R Pathan

**Affiliations:** 1 Pediatric Cardiac Surgery, Bhanubhai and Madhuben Patel Cardiac Centre, Bhaikaka University, Anand, IND; 2 Pediatric Cardiac Surgery, Sri Padmavati Pediatric Heart Centre, Sri Venkateswara Institute of Medical Sciences (SVIMS) Campus, Tirupati, IND; 3 Radiodiagnosis and Imaging, Pramukhswami Medical College and Shree Krishna Hospital, Bhaikaka University, Anand, IND; 4 Pediatric Cardiac Intensive Care, Bhanubhai and Madhuben Patel Cardiac Centre, Bhaikaka University, Anand, IND; 5 Department of Pediatrics, Pramukhswami Medical College and Shree Krishna Hospital, Bhaikaka University, Anand, IND; 6 Cardiac Anaesthesiology, Bhanubhai and Madhuben Patel Cardiac Centre, Bhaikaka University, Anand, IND; 7 Clinical Research Services, Bhanubhai and Madhuben Patel Cardiac Centre, Shree Krishna Hospital, Bhaikaka University, Anand, IND

**Keywords:** polytetrafluoroethylene (ptfe), conduits, homograft, allograft, truncus arteriosus

## Abstract

Although a new right ventricle outflow can be introduced during pulmonary artery reconstruction, it is a suboptimal option as the valved conduits that mimic the natural right ventricular outflow do not grow, and a surgical conduit replacement cannot be averted. This study reported the implementation of hand-made polytetrafluoroethylene (PTFE) tricuspid-valved conduits to rebuild the right ventricular outflow tract in toddlers with truncus arteriosus and risk factors for earlier conduit explant. Herein, we described a case report of a 9-month-old toddler diagnosed in November 2021 with truncus arteriosus type I with ventricular septal defect (VSD) and severe pulmonary arterial hypertension, who has been successfully discharged 20-days postoperative surgical reconstruction with good bi-ventricular functions. Hand-made PTFE tricuspid-valved conduits are efficient in the reconstruction process of the right ventricular outflow tract in children with truncus arteriosus. The conduits are cheap, easily available, and lack potential sensitization.

## Introduction

McGoon, Rastelli, and Ongley, in 1965, performed the first successful correction of the truncus arteriosus using a valved aortic allograft for establishing continuity between the right ventricle and the pulmonary artery [1]. Various modifications and improvements have been suggested since then, with an unequivocal tendency toward performing the primary repair as early as possible to eschew possible complications like irreversible pulmonary arterial hypertension [2,3]. However, some challenges persist, such as managing the related lesions or different anatomical subgroups as well as choosing the optimal approach to create a stable connection from the right ventricle to the pulmonary artery. The ideal method for right ventricular outflow tract (RVOT) reconstruction in neonatal truncus repair is through an allograft-valved conduit, that is because of its adequate hemodynamic profile and tissue processing properties [2,3]. Allografts availability for early infants is limited, particularly in terms of sizes that can be used in surgery. To overcome these obstacles and reduce the surgical procedure risks, xenograft with valved conduits and Dacron porcine conduits are considered reasonable alternatives [4]. However, the rate of re-intervention due to early obstruction is higher with the latter than with allograft [5]. Therefore, in early 1990, Barbero-Marcial et al. intended to use autologous tissue reconstruction of RVOT for altering the common arterial trunk [6]. They assembled a direct link uniting the right ventricle and pulmonary artery using a bovine pericardial monocusp valve. This method prevented the use of conduits, thereby making theoretical growth possible and subsequent excessive surgical interventions. However, various concerns have been raised concerning the durability and functional characteristics of pericardial monocusp valves [7].

A polytetrafluoroethylene (PTFE) monocusp outflow patch technique, proposed by Brown et al., provided a functioning pulmonary valve [8]. Efforts to produce safe, long-lasting, and cost-efficient homograft alternatives are still ongoing. Our institution employs hand-made tricuspid-valved PTFE conduits using basic-stretch PTFE grafts and microporous PTFE membranes for RVOT reconstruction. In this study, we aimed to examine the implementation of these PTFE tricuspid-valved conduits and define potential risk factors for lessened conduit durability in toddler patients.

## Case presentation

Written informed consent was taken from the parents before surgical reconstruction was initiated. A detailed cardiological evaluation of the patient was performed before cardiac surgery via 2-D echocardiography and cardiac computed tomography (CT) dynamic scan (Table 1).

**Table 1:** Patient characteristics at the time of conduit grafting BSA: body surface area

Age (months)	9
Weight (kg)	5.0
Sex	Female
Height (cm)	59
BSA (m^2^)	0.29
Birth weight (kg)	2.9
Preoperative oxygen saturation (%)	96
Cyanotic spells	Nil
Previous surgical procedure	Nil
Diagnosis	Truncus arteriosus type I/A I
McGoon ratio	1.96
Nakata index (mm^2^/m^2^)	483.57
Blood group	O Rh-positive

Preoperative 2-D echocardiography unveiled truncus arteriosus (type I) in addition to the right aortic arch and a large subtruncal ventricular septal defect (VSD) shunting bi-directional from both ventricles. No additional VSDs were observed. Immediately after the common truncal valve, the ascending aorta and main pulmonary artery arise from the common trunk. The main pulmonary artery and branch pulmonary arteries were appropriately sized. No atrial septal defect was detected. We found mild tricuspid valve regurgitation but no mitral valve regurgitation. Although quadricuspid truncal valve and mild regurgitation were noted, no stenosis was observed. The right atrium and ventricle, as well as the left atrium, were dilated. The aortic arch was right-sided with mirror-image branching of neck vessels.

**Figure 1:**
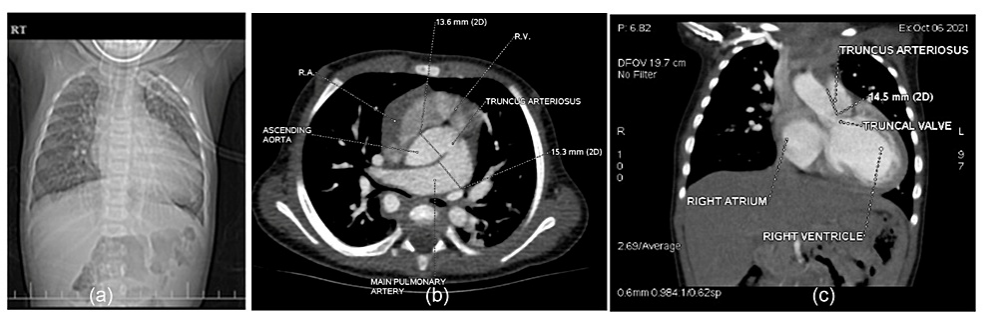
(a) Scannogram of chest AP: widened superior mediastinum with plethoric lung fields; (b) contrast-enhanced cardiac CT axial image: truncus arteriosus C & E type 1 and Van Praagh type A1; (c) contrast-enhanced cardiac CT coronal image: truncus arteriosus C & E type 1 and Van Praagh type A1 with common trunk having a length of 14.5 mm

**Figure 2:**
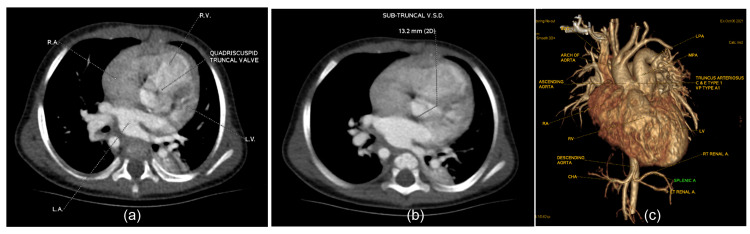
(a) Contrast-enhanced cardiac CT axial image: quadricuspid truncal valve; (b) contrast-enhanced cardiac CT axial image: sub-truncal ventricular septal defect (VSD) of 13.2 mm size; (c) volume rendering (VR) anterior view: truncus arteriosus C & E type 1 and Van Praagh type A1. Right aortic arch giving four branches from anterior to posteriorly—left subclavian artery, left common carotid artery, right common carotid artery, and right subclavian artery. Common hepatic artery arising from superior mesenteric artery and coeliac trunk continuing toward the left side at splenic artery

A cardiac CT dynamic study confirmed the diagnosis of truncus arteriosus (Collett and Edwards classification and Van Praagh classification, type I) measuring 14.5 mm in length and a big subtruncal VSD measuring 13.2 mm in diameter. The right-sided aortic arch with separate origin of left subclavian artery, left common carotid artery, right common carotid artery, and right subclavian artery from the arch of aorta (from anterior to posterior), as well as the immediate origin of right vertebral artery from the right subclavian artery, were observed. The cardiac CT scan also established the non-visualization of the thymus, possibly absent or hypoplastic, as well as both plethoric lung fields (Figures 1-2).

After optimizing the preoperative workup, including molecular analysis for the qualitative detection of SARS-CoV2 (which turned out to be negative), the patient was posted for corrective surgery: Truncus repair from the right ventricle to pulmonary artery 14 mm, PTFE Gore-Tex hand-made tricuspid-valved conduit was used and glutaraldehyde-treated pericardial patch closure of the VSD was done (Table 2).

**Table 2:** Operative data CPB: cardiopulmonary bypass; ACC: aortic cross-clamp; PTFE: polytetrafluoroethylene

CPB time	242 min
ACC time	185 min
Cardioplegia solution	Del Nido crystalloid cardioplegia
Conduit size	PTFE 14 mm

As the thymus was unobservable in the cardiac CT dynamic study and there was no emergency, an irradiated blood strategy was adopted for the patient, and the chance of severe immunodeficiency co-existing with DiGeorge or CHARGE syndromes was only 0.5%-1% [9,10].

As per British guidelines, in neonates and infants with suspected T cell deficiency, T cell lymphocyte count should be performed pre-operatively. If the T cell count is >400 cells/µl (of which 30% are naive T cells), irradiated blood is not required. Likewise, in children aged >2 years with suspected DiGeorge syndrome but without significant infection history, there is no need for irradiated blood. The Canadian Transfusion Guidelines advise irradiated blood transfusion in neonates with complex cardiac anomalies if immunodeficiency defects, such as the DiGeorge syndrome, are not excluded; in infants, irradiated blood transfusion is recommended only when immunodeficiency is suspected.

Polytetrafluoroethylene conduit construction

We used a PTFE-valved conduit prepared in the operating room before the cardiac procedure of the 9-month-old female baby (weight 5 kg) to reduce operative time.

Based on previous nomograms for pulmonary valve annulus size, the Z-scores determined the conduit size taking into account the conduit diameter and body surface area. For example, a patient weighing 5 kg requires a 7.5-mm pulmonary annulus and 5.5-mm branch pulmonary artery (as per the minimum acceptable pulmonary valve ring diameter employed by Kirklin in 1975, 1976).

**Figure 3:**
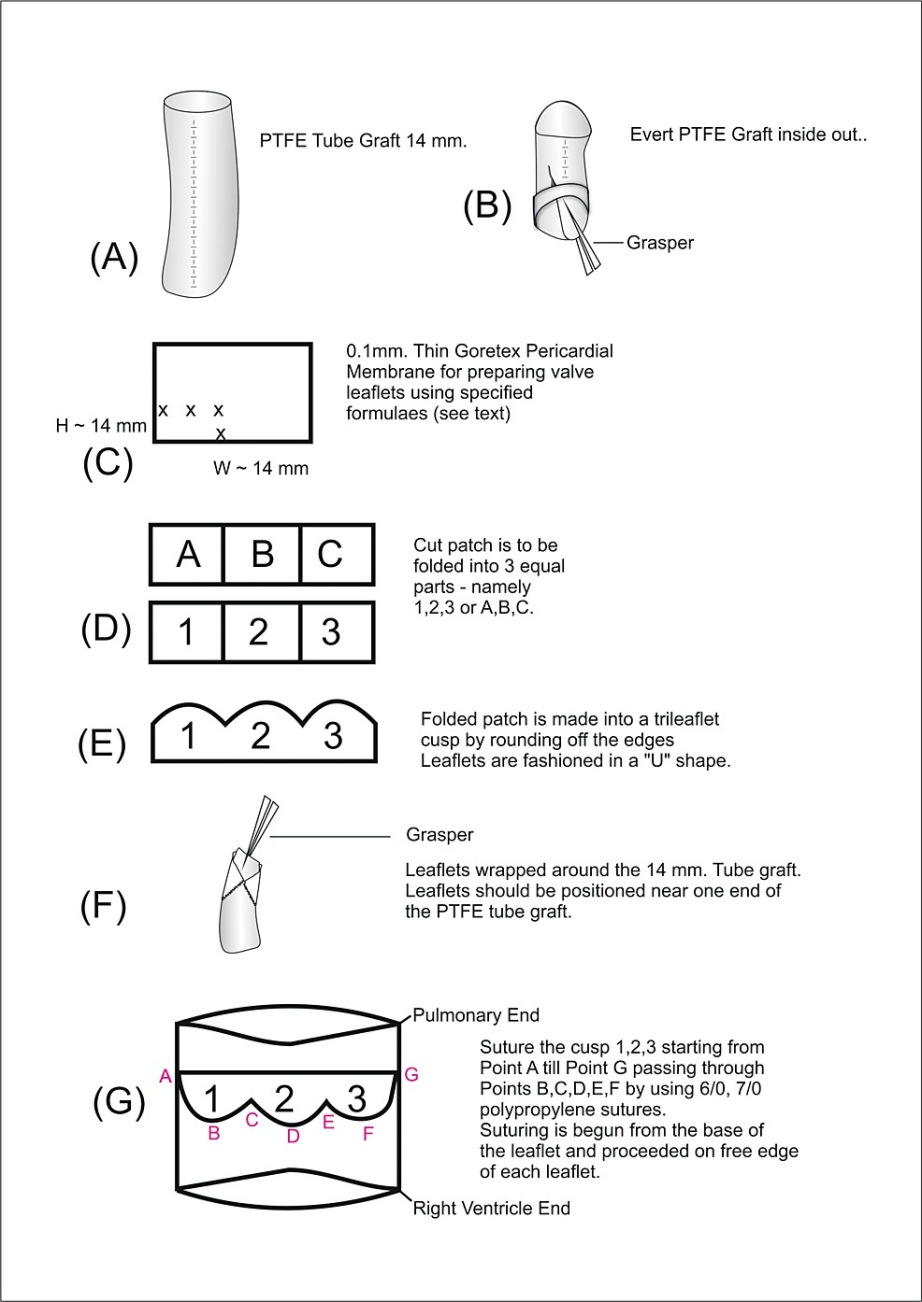
Method of PTFE trileaflet-valved conduit construction PTFE: polytetrafluoroethylene Image Credits: Dr. Vishal V. Bhende

**Figure 4:**
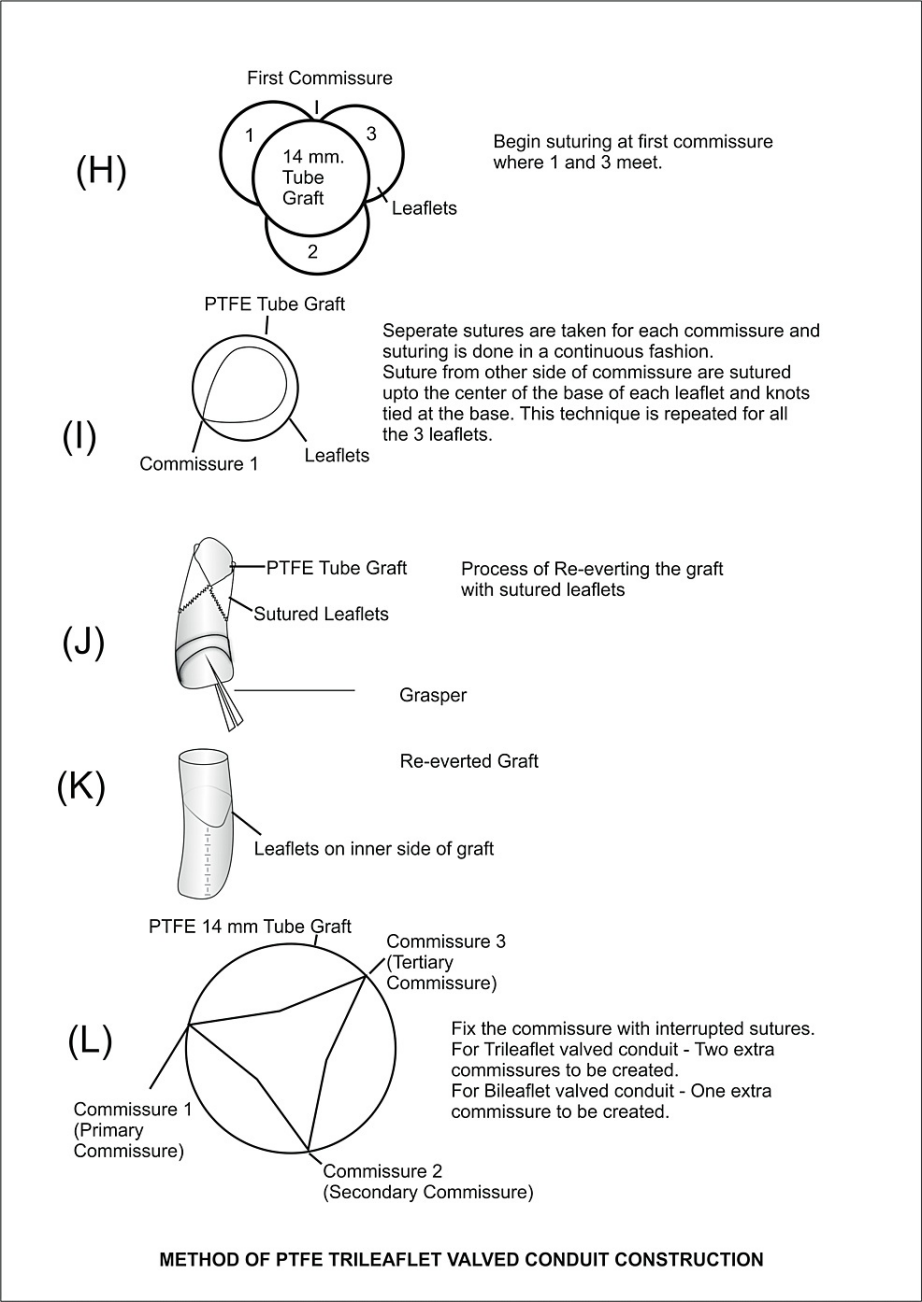
Method of PTFE trileaflet-valved conduit construction PTFE: polytetrafluoroethylene Image Credits: Dr. Vishal V. Bhende

Considering the attrition factor involved in the conduits, we chose a 14-mm PTFE tube graft (Figures 3-4).

The following formulae were used to prepare the conduits:

1. Height of the leaflet = 0.75 × graft diameter + 1-2 mm.

2. The diameter of the valve in millimeters is used as the height and width of the leaflet.

Median sternotomy was performed under general anesthesia with the monitoring lines in place. The thymus was dissected, and the pericardium was cleared adequately for the patch and then marsupialized. Then, the patient was heparinized. We performed a bypass using aortic and selective bicaval cannulation techniques after snaring the main pulmonary artery (MPA) emerging from the trunk (Table 3).

**Table 3:** Surgical summary RVOT: right ventricular outflow tract; PTFE: polytetrafluoroethylene

1.	The large subtruncal ventricular septal defect (VSD) was closed by a glutaraldehyde-treated pericardial patch.
2.	Main pulmonary artery (MPA) transected from the aorta.
3.	Aorta-pulmonary communication closed by a glutaraldehyde-treated pericardial patch.
4.	The right ventricle opened on the anterior wall while avoiding injury to major coronary artery branches.
5.	Obstructing muscle bundles excised.
6.	Hand-made PTFE/ Gore-Tex tube conduit (14 mm) fashioned with a trileaflet valve using a 0.1-mm PTFE membrane.
7.	Conduit anastomosed to RVOT augmented with a Synkroscaff (bovine pericardial patch).
8.	Conduit distal anastomosis was done to MPA -Left pulmonary artery (LPA) confluence using 6/0 polypropylene continuous sutures.

**Figure 5:**
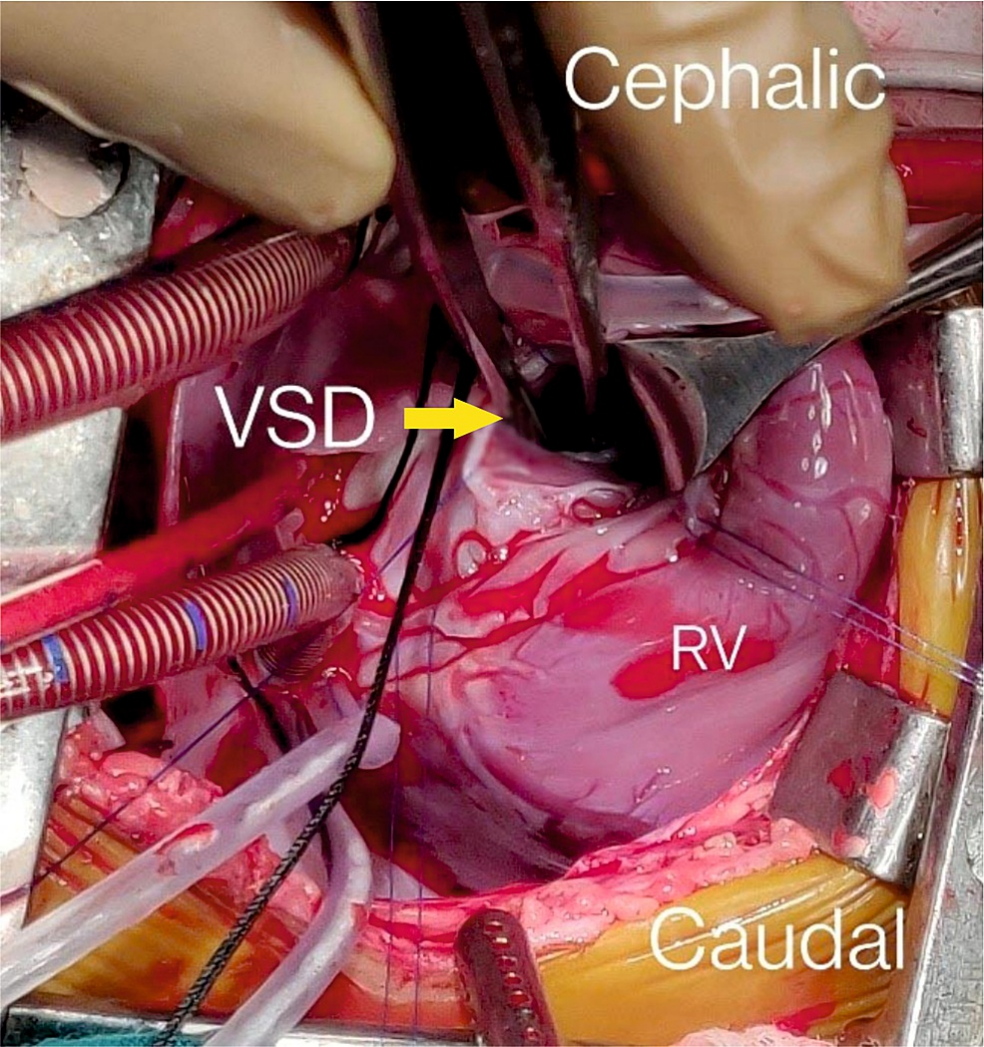
VSD repair with a glutaraldehyde-treated pericardial patch VSD: ventricular septal defect; RV: right ventricle

**Figure 6:**
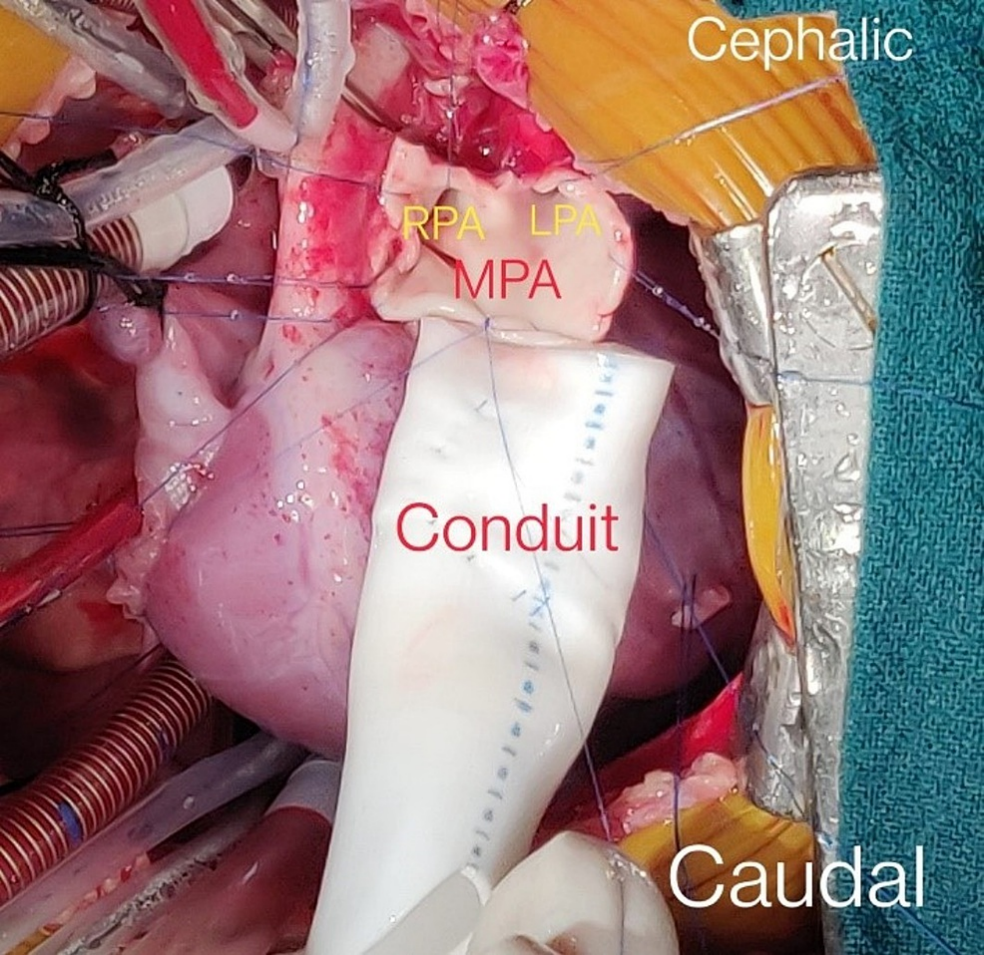
Conduit anastomosed to right ventricular outflow tract (RVOT) RPA: right pulmonary artery; LPA: left pulmonary artery; MPA: main pulmonary artery

Large subtruncal VSD closed with glutaraldehyde-treated pericardial patch (Figures 5-6) (Video 1).

**Video 1:** Truncus arteriosus repair with hand-made PTFE-valved conduit

The patient was operated on uneventfully, and her chest was left open and covered with a blood bag. Delayed chest closure was performed on postoperative day 2 (Table 4).

**Table 4:** Postoperative data POD: Postoperative day

Delayed chest closure	POD 2
Ventilator hours	335 hours (13 days)
Length of ICU stay	17 days
Length of hospital stay	22 days

The patient had grown two organisms in her endotracheal secretions 48 hours after incubation, *Candida albicans* and *Staphylococcus aureus* (methicillin-resistant *S. aureus* (MRSA))-which were treated with sensitive antibiotics.

**Figure 7:**
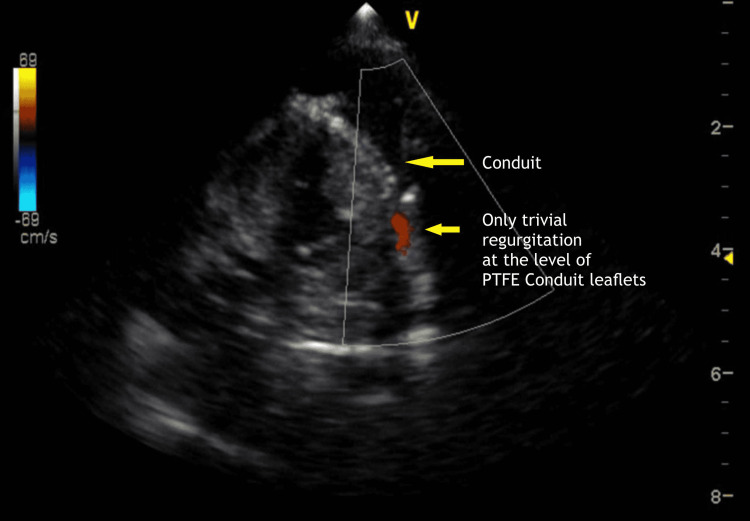
Postoperative 2D echocardiography showing trivial pulmonary regurgitation across the PTFE conduit leaflet PTFE: polytetrafluoroethylene

2-D echocardiography at discharge revealed a residual VSD measuring 4 mm in 2D, shunting left to right, with unobstructed flow from the right ventricle to the pulmonary artery through the conduit, trivial pulmonary regurgitation, moderate tricuspid valve regurgitation with the peak gradient 48 mmHg as well as moderate pulmonary arterial hypertension (Figure 7).

## Discussion

PTFE is a biologically inert, non-immunogenic material that is fairly economical; raw materials of PTFE-valved conduits cost one-tenth of similar size pulmonary homograft conduits. Prosthetic valves made of PTFE have been used since the beginning of the 1990s. On the other hand, Brown et al. described excellent lengthy-term results of the use of PTFE as a monocusp valve in RVOT; it diminished initial and midterm pulmonary insufficiency without significant stenosis [8]. The practice has indicated that causes like stenosis and insufficiency require, especially in toddlers and little children, the spare of almost all conduits employed for RVOT reconstruction using cryopreserved homografts; however, young patient’s age and small conduit size are risk factors for low conduit durability [8,11-14]. This fact, along with factors such as suboptimal performance, high cost, limited availability, and potential for immunogenicity of these conduits, has led to the development of alternate prosthetic conduits, including Porcine Xenografts, Dacron conduits, Contegra (Bovine jugular venous valve, Medtronic, Minneapolis, MN, USA), as well as valved and unvalved PTFE tubes [15-17]. In contrast, Quintessenza et al. have successfully constructed PTFE valves in RVOT in toddlers and young adults and have recently adapted their approach to incorporating them within PTFE conduits [18].

Ando and Takahashi described in the study their conclusions after using trileaflet PTFE valves for more than a decade within Dacron conduits. They reported 88 % freedom from conduit explantation at 10 years [19].

Miyazaki et al. developed valved PTFE conduits because homograft conduits are not widely available in Japan and Yamashita et al. categorized them in various forms, such as bicuspid, tricuspid, with, and without bulging sinuses, and are widely used in Japan, and promising midterm outcomes have been reported [20,21]. Shinkawa et al. compared valved PTFE conduits with other alternatives, revealing 92.1% freedom from conduit reoperation at 5 years, which is a significant improvement compared with pulmonary homografts, which have 76.8% freedom [22].

In our previous experience, trileaflet-valved conduits are preferable to bileaflet valved conduits because they are more physiologic, allowing for a more thorough coaptation of the valve leaflets. As a result of the impetus provided by our Japanese colleagues, the inclusion of bulging sinuses might be a significant and valuable adjustment to our current technique.

Conduit replacement cannot be avoided in infants and young children due to the inherent somatic conduit outgrowth; however, some other factors lead to adverse outcomes or the necessity of earlier reoperation, such as valve incompetence or stenosis.

Regardless of somatic outgrowth, conduit upsizing is recommended when the z-score is greater than +2 or between +1 and +3. Wells et al. found that only 8% of 40 patients experienced somatic outgrowth and given that oversized conduits can cause external compression and earlier repair, they recommended initial conduit placing with a z-score of 0 [23].

Considering their conclusions and the fact that stenosis is the main reason for conduit exchange, we agree with conduit upsizing at implant when feasible.

From recent literature, we have gathered the main causes for the failure of PTFE conduits:

1) Shrinkage or calcification of PTFE grafts.

2) Over time, the distal anastomosis develops fibrosis and narrows (in the case of valved PTFE conduits).

3) No fibrous tissue is evident in the conduit.

4) Stenosis of the valve leaflets due to fibrosis.

5) Leaflets “stuck” in the open position, leading to conduit insufficiency.

In the Indian subcontinent, where low- and middle-income group populations outnumber others, the management of cost-effective medical supplies becomes imperative.

PTFE conduits of 14 mm and 20 cm in length cost approximately 50,000 INR (663.58 US$; maximum retail price (MRP). As they fall under the category of “stretched vascular grafts,” the required length of implants can be tailor-made for individual patients, which further reduces their costs.

(10 cm: 25,000 INR (331.79 US$); 5 cm: 12,500 INR (165.90 US$).

PTFE conduits require Gore-Tex pericardial membrane for in-built valve preparation. One can prepare a bileaflet or trileaflet valve using this membrane. The cost of a 0.1-mm Gore-Tex pericardial membrane is Rs. 46,000 INR for 6 × 12 cm (610.50 US$).

When the total PTFE conduit cost is calculated, including costs of the PTFE tube and Gore-Tex pericardial membrane, the result is about one-third of the commercial price of bovine jugular vein conduits (Contegra).

Contegra conduits are available in sizes of 12-18 mm and cost approximately INR 1,50,000 + 12% Goods & Services Tax (GST) = 1,68,000 INR (2206 US$). Hospitals have to incur these costs as they are not covered by government scheme packages. Alternative conduits include pulmonary homografts available at certain institutions that have access to homograft banks and Dacron conduits. Pulmonary homografts must be harvested and preserved in the homograft banks and can be used free of cost by the specific institutions which harbor these banks. They are only sometimes sold to other institutions for a price depending upon the homograft license available at the recipient institution. Dacron conduits are also sometimes used albeit rarely as they are usually available in size 14 mm. Dacron tubes of 30 cm in length cost approximately 35,000 INR (464.51 US$).

After the repair of truncus arteriosus at any institution, the facility of inhaled nitric oxide (iNO) is kept available, especially in the postoperative period, as iNO is commonly used as anti-pulmonary hypertensive medication after truncus arteriosus repair. As it is delivered via the inhalational route, it acts exclusively on pulmonary circulatory vessels. It activates soluble guanyl cyclase, thereby helping in dilating pulmonary arteries. The administration of iNO usually begins in the operating room and continues for the initial 1-7 postoperative days. As its routine dose has no systemic effect, it is widely used after truncus repair; a multicenter study estimated its use at 46% in patients requiring truncus repair [24]. However, its use is center-dependent as low-volume centers use it more frequently.

## Conclusions

We reported the case of a 9-month-old child with truncus arteriosus type I, which was repaired using valved PTFE conduits. A bicuspid and tricuspid-valved PTFE conduits performed similarly to bovine pericardial conduits, which are rare and expensive. Upon review of the literature, it was found that there are no significant differences in the time it takes for conduit stenosis or insufficiency to develop between the various classes. A risk factor for extra conduit implantation is the young age at the time of surgery; however, a larger conduit z-score prevents the development of conduit stenosis. Therefore, valved PTFE conduits can be used instead of homograft conduits because of availability, ease of construction, in addition to low cost and sensitization risk. Considering the Indian scenario and the longevity of conduit vis-à-vis conduit attrition and cost-effectiveness, PTFE is a handy and effective option in limited-resource environment settings.
